# Delivering psychological care in palliative oncology: The case for counseling competency within interdisciplinary nursing interventions

**DOI:** 10.1017/S1478951526102910

**Published:** 2026-06-11

**Authors:** Ade Herdian Putra, Zadrian Ardi, Evi Afiati

**Affiliations:** 1 Department of Guidance and Counseling, Universitas Negeri Padang, Padang, Indonesia; 2 Department of Guidance and Counseling, Faculty of Teacher Training and Education, Universitas Sultan Ageng Tirtayasa, Serang, Indonesia

The study by Ibrahim and Zaghamir (2026) demonstrates that a structured 6-week nurse-led palliative intervention can significantly improve quality of life, reduce distress, and lower symptom burden in older adults with cancer. The intervention’s multidimensional design, addressing physical, social, functional, and spiritual domains simultaneously, represents a genuinely integrative model of palliative care. We write to raise a question that the study invites but does not address: what level of training and competency is required to deliver the psychological components of such an intervention responsibly and effectively?

Sessions 3 through 6 of the program include cognitive-behavioral techniques, mindfulness strategies, emotional expression facilitation, coping with uncertainty, and spiritual counseling. These are structured psychological interventions that, in most clinical and research contexts, require specialized training in psychotherapy or counseling to deliver safely and with fidelity. The palliative care literature consistently recognizes that psychological care exists on a continuum of complexity requiring differentiated professional competencies. The National Institute for Health and Care Excellence model distinguishes between general psychological support deliverable by any team member and formal therapeutic interventions requiring trained mental health professionals, specifically psychologists, psychiatrists, or counselors (Feldstain 2024). Ibrahim and Zaghamir do not describe the specific training their nurses received in Cognitive Behavioral Therapy (CBT), mindfulness delivery, or spiritual care facilitation, nor do they clarify whether any sessions were supervised by qualified mental health professionals.

This matters for 2 reasons. First, it limits replicability. Clinicians seeking to implement this program cannot determine whether outcomes are attributable to session structure, nurses’ general relational skills, or specific therapeutic competencies not routinely available in oncology settings. Second, it raises an ethical question. Cognitive-behavioral and spiritual interventions delivered without adequate training risk superficial engagement with complex psychological material, or unintentional harm through poorly managed emotional disclosure in patients near the end of life (Brenner et al. 2021).

We propose that future iterations of this intervention explicitly incorporate guidance and counseling professionals as co-facilitators or supervisors of the psychological components. Interdisciplinary models in which oncology nurses manage physical and functional domains while trained counselors or psycho-oncologists lead psychological and spiritual sessions have been shown to improve both the quality and safety of palliative psychological care (Feldstain 2024). Reporting the professional background and training of all intervention facilitators as a standard element of palliative intervention studies would also strengthen the evidence base and support responsible implementation across diverse clinical settings.
